# Assessment of Well-Being Across Menstrual Phases in Female Students

**DOI:** 10.1089/whr.2023.0033

**Published:** 2023-10-31

**Authors:** Hiroe Washio

**Affiliations:** School of Nursing, Takarazuka University, Osaka, Japan.

**Keywords:** female nursing student, menstrual cycle, autonomic nervous activity, follicular phase, luteal phase

## Abstract

**Purpose:**

In this study, we determined female nursing students' physical and mental state involvement by measuring heart rate variability and salivary α-amylase activity (αAMY).

**Methods:**

The study included 108 students aged 20–21 with regular menstrual cycles. The basal body temperature method was used to determine the menstrual phases. Five indices were used: *low* and *high frequency components*, and their *ratio*, *total power*, and *physical stress index*. In addition, αAMY was measured using a salivary amylase monitor. A six-point scale was used for subjective mood evaluation. Repeated measures analysis of variance was performed for differences between groups, and the Tukey–Kramer method was used for multiple comparisons. A *p*-value of <0.05 was considered statistically significant.

**Results:**

The results suggested that the physical fatigue of the luteal phase is carried over into the menstrual phase, and the symptoms concomitant with the menstrual phase may manifest as poor mood. Furthermore, parasympathetic activity and stress coping skills tended to be higher, and sympathetic activity was lower during the menstrual phase, suggesting that students are more relaxed during the menstrual period.

**Conclusion:**

Though the results were statistically not significant, the students were in a considerably better mood during the follicular phase than the menstrual phase, suggesting that the physical and mental states may differ between the early and late follicular phases. It may be possible to evaluate the mental and physical condition of female students by obtaining more values.

## Introduction

In recent years, autonomic nervous system assessment has been recognized as one of the indicators of stress evaluation, with an analysis made possible by noninvasive heart rate variability (HRV) measurement. For example, in a study of 22 female college students, females had higher parasympathetic and lower sympathetic activity than males.^1^ According to another study of 30 females aged 22–37, sympathetic nerve activity and arrhythmias increased during the luteal phase.^2^ Autonomic Nervous Activity (ANA) assessment was done primarily through HRV measurement, which classified the menstrual cycle into two phases: follicular and luteal. These studies suggest that the most challenging times for students are the premenstrual and menstrual periods (menstruation with premenstrual syndrome and dysmenorrhea).

The menstrual cycle can be classified on the basis of the presence of hormonal patterns and its effect on various female organs. This study classified the menstrual cycle into three phases: menstrual, follicular, and luteal. HRV and salivary alpha-amylase activity (αAMY), an index of exchange nerve activity, were measured to evaluate the mental and physical states of female nursing students involved in preserving human life and to help them maintain normal states.

## Materials and Methods

### Research participants and design

The study included female undergraduate nursing students aged 20 to 21 years who were on the verge of entering nursing practice and had consented to participate in the study. The participants had menstrual cycles of 25 to 38 days, within the normal range. One hundred eight participants were included in the study, excluding those with underlying diseases and those taking medications. Since protein, fat, sucrose, and coffee increase αAMY and sake (Japanese rice wine) suppresses it^3^; the participants were forbidden from consuming caffeine, smoking, drinking alcohol, and eating and drinking anything other than water for 1 hour before the tests.

The tests were conducted in the morning as αAMY fluctuates throughout the day.^4^ αAMY and HRV measurements were conducted consecutively in a private room. The measurement of blood hormone levels is accurate for determining each phase of the menstrual cycle. However, studies on body water content^5^ and caloric intake^6^ have used the basal body temperature method. The day when menstrual bleeding began to the day before it disappeared was defined as the menstrual period. The period beginning the day after menstruation ended and extending up to 2 days before the conclusion of the low-temperature phase of the basal body temperature was designated as the follicular phase, while the period starting from the third day of the high-temperature phase of the basal body temperature was designated as the luteal phase. The Clinical Trial Registration number is UMIN000023537.

### Ethical considerations

The study was conducted according to the modified version of the Declaration of the Helsinki Creed of 2013 and with the approval of the Ethics Committee of Kio University (Approval No. H29-02-1). Oral informed consent was obtained after the study participants received an explanation on the following items: the purpose of this research; research cooperation is voluntary; no disadvantage occurs even if one chooses not to participate or withdraws during the research; results will not be used for any purpose other than the research; and the management of the research will be conducted in such a way that names cannot be identified.

### Physical measurements

Height, weight, body mass index, body temperature, and blood pressure were measured during each visit.

### Heart rate variability

Measurements were made using the Pulse Analyzer Plus TAS9 (YKC Corporation, Japan). During the perinatal period, parasympathetic activity is increased primarily by oxytocin.^7^ The device used in the study was designed to analyze ANA using accelerated pulse waves obtained from the fingertips. In this study, five indices of ANA were analyzed: low frequency (LF), high frequency (HF), LF/HF ratio, total power (TP = HF + LF + very low frequency [VLF]), and physical stress index (PSI). A decrease in LF indicates acute stress or fatigue, whereas HF reflects parasympathetic activity. The LF/HF ratio reflects sympathetic activity, and TP reflects stress-coping ability. In addition, PSI reflects the degree of physical fatigue. The participants remained at rest in a half-seated position for ∼900 seconds both before and during the measurements.

### Measurement of αAMY

The digestive enzyme αAMY is an index for acute stress based on sensitivity to stimuli with a response time of 60 to a few seconds.^8^ A salivary amylase monitor (Nipro Corporation, Japan) that measures the stress state by measuring the increase or decrease in sympathetic nerve activity was used.^9^ The tip of the filter paper was placed under the tongue for about 30 seconds, and saliva was collected and measured.

### Measurement of subjective mood

Subjective mood was assessed using a six-level scale. The six levels are 0 (*feeling very good*), 1 (*feeling fairly good*), 2 (*not feeling good*), 3 (*feeling bad*), 4 (*feeling pretty bad*), and 5 (*feeling very bad*). The good and bad mood scores were 0–1 and 2–5, respectively.

### Statistical analyses

All values are expressed as mean ± standard deviation. Differences between groups were analyzed with repeated measures of analysis of variance, and multiple comparisons were performed using the Tukey–Kramer method. Furthermore, all statistical analyses were performed using the Excel Statistics software (Excel 2012 version 1.14 for Windows; Social Survey Research Information Co. Ltd., Tokyo, Japan). *p* < 0.05 was considered statistically significant. A normality test for the data was performed.

## Results

### Research participants

The characteristics of the 108 female nursing college students at each menstrual phase are illustrated in Table 1. No statistically significant difference was detected between each menstrual phase and the participants' measurements.

**Table 1.** Characteristics of Participants

	Follicular phase	Luteal phase	Menstrual phase
Age (years)	20.6 ± 0.9	20.6 ± 1.1	20.2 ± 1.0
Height (m)	1.599 ± 0.063	1.589 ± 0.054	1.605 ± 0.059
Body weight (kg)	56.5 ± 7.6	54.3 ± 8.2	56.9 ± 8.9
Body mass index (kg/m^2^)	21.9 ± 2.6	22.4 ± 2.6	21.9 ± 2.8
Body temperature (K)	309.65 ± 273.45	309.75 ± 273.45	309.55 ± 273.55

Each value is expressed as mean ± standard deviation.

### Heart rate variability

**FIG. 1.**
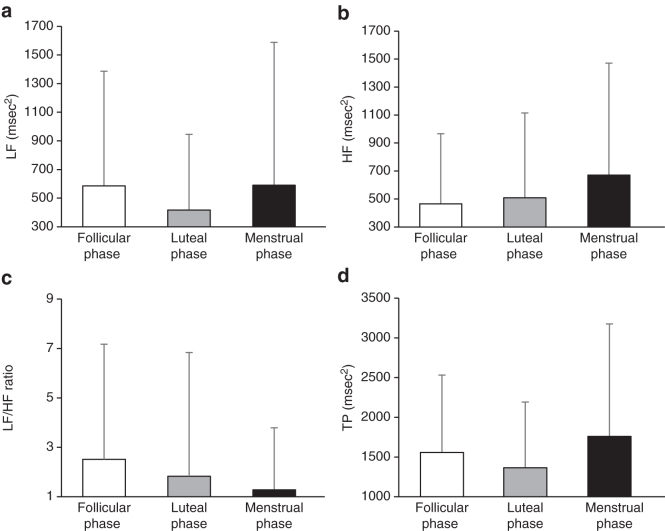
Autonomic nerve activity indices during each phase of the menstrual cycle (follicular phase, luteal phase, and menstrual phase) **(a)** LF; **(b)** HF; **(c)** LF/HF ratio; **(d)** TP. HF, high frequency; LF, low frequency; TP, total power.

**FIG. 2.**
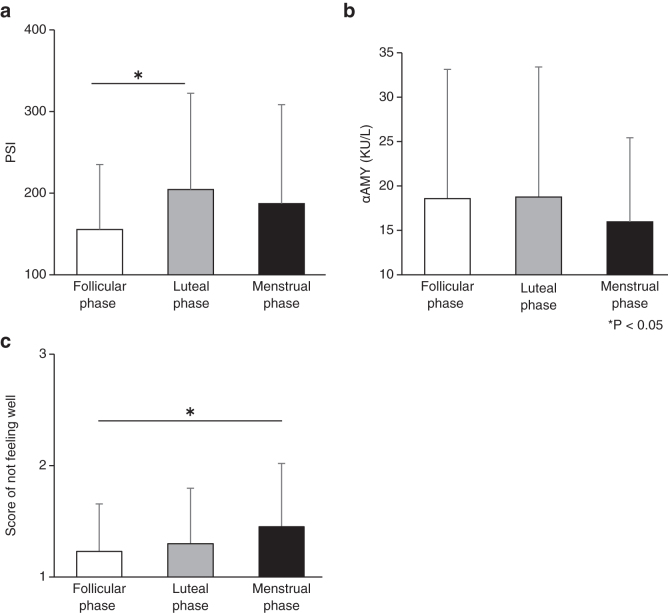
Autonomic activity indices during each menstrual phase (follicular phase, luteal phase, and menstrual phase) **(a)** PSI; **(b)** αAMY; **(c)** Score of not feeling well. αAMY, α-amylase activity; PSI, Physical stress index.

The mean and standard deviation of LF, an HRV index of the participants, were 585.8 ± 801.7, 417.0 ± 528.5, and 589.8 ± 998.8 msec^2^ for the follicular, luteal, and menstrual phases, respectively. The HF was 468.9 ± 499.7, 512.2 ± 605.3, and 674.0 ± 798.1 msec^2^ for the follicular, luteal, and menstrual phases, respectively. The LF/HF ratio was 2.5 ± 4.7, 1.8 ± 5.0, and 1.3 ± 2.5 for the follicular, luteal, and menstrual phases, respectively. The TP of the participants was 1561.9 ± 972.9, 1369.5 ± 825.9, and 1762.3 ± 1413.8 msec^2^ for the follicular, luteal, and menstrual phases, respectively. The PSI was 155.5 ± 79.6, 204.4 ± 118.0, and 187.3 ± 121.1 in the follicular, luteal, and menstrual phases, respectively. The PSI was significantly higher in the luteal phase than in the follicular phase. The follicular phase tended to have lower HF and higher LF/HF ratios than the menstrual and luteal phases. However, LF, HF, LF/HF ratio, and TP were not significantly different among the menstrual phases. On the contrary, LF and TP were lower, and PSI was higher in the luteal phase than in the follicular and menstrual phases. In the menstrual phase, HF and TP tended to be higher. In the menstrual phase, LF/HF ratio tended to lower than in the follicular and luteal phases (Figs. 1 and 2).

### α-Amylase activity

The mean and standard deviation of αAMY of the participants were 18.6 ± 14.6, 18.7 ± 14.7, and 16.0 ± 9.5 KU/L in the follicular, luteal, and menstrual phases, respectively. There were no significant differences among the phases. However, the average values during the menstrual phase tended to be lower than those during the follicular and luteal phases (Fig. 2).

### Subjective mood

The percentages of the poor mood scores were 23.1% (12 out of 52), 31.6% (18 out of 57), and 48.4% (15 out of 31) in the follicular, luteal, and menstrual phases, respectively. The poor mood score percentage was significantly higher in the menstrual phase and significantly lower in the follicular phase (Fig. 2).

## Discussion

This study demonstrated no significant differences in the LF/HF ratio or αAMY for sympathetic activity, HF for parasympathetic activity, and LF for stress or fatigue in the follicular, luteal, or menstrual phases. Parasympathetic and sympathetic activity predominate in the follicular and luteal phases, respectively.^2^ Additionally, αAMY serves as an indicator of acute stress in response to stimuli, exhibiting a rapid response within one to several minutes.^8^ Furthermore, stress and sympathetic activity correlate positively.^10^ Therefore, the results obtained in this study did not coincide with those of previous studies.

This study classified the menstrual cycle into three phases. In this study, physical fatigue was significantly higher in the luteal than in the follicular phase, suggesting that, in the luteal phase, sympathetic nervous activity is significant and stressful, leading to physical fatigue. Furthermore, the percentage of women who felt worse during the menstrual phase was significantly higher than those who felt worse during the follicular phase. In other words, it was demonstrated that many female students feel sick during the menstrual phase. Physical fatigue from the luteal phase persists in the menstrual period, and accompanying symptoms may be responsible for poor mood. Although not significantly different, parasympathetic nervous activity (HF) and stress coping skills (TP) were higher during the menstrual phase compared to the luteal and follicular phases. Sympathetic nervous activity (LF/HF ratio, αAMY) tended to be lower, suggesting that many participants were relaxed during the menstrual phase.

In addition, the participants experienced significantly better moods during the follicular phase than during the menstrual phase, suggesting that physical and mental states may differ between the early and late phases of the follicular phase. The menstrual cycle can be classified on the basis of the presence of hormonal patterns and its effect on various female organs. More research participants should be recruited to examine the relationship between HRV in each menstrual cycle.

## Data Availability

All data generated or analyzed during this study are included in this published article.

## Abbreviations Used

αAMY: α-amylase activity
ANA: Autonomic Nervous Activity
HF: high frequency
HRV: heart rate variability
LF: low frequency
PSI: physical stress index
TP: total power
VLF: very low frequency
